# Oviposition and Larval Habitat Preferences of the Saltwater Mosquito, *Aedes vigilax*, in a Subtropical Mangrove Forest in Queensland, Australia

**DOI:** 10.1673/031.012.0601

**Published:** 2012-01-24

**Authors:** Jon Knight, Lachlan Griffin, Pat Dale, Stuart Phinn

**Affiliations:** ^1^ Australian Rivers Institute, Griffith School of Environment, Griffith University, Nathan, Queensland, Australia, 4111; ^2^ School of Geography, Planning and Environmental Management, University of Queensland St. Lucia, Queensland,Australia, 4072; ^3^ Environmental Futures Centre, Griffith School of Environment, Griffith University, Nathan, Queensland, Australia,4111

**Keywords:** basin forest, Coombabah Lake, depression, eggshell density, environmental characteristics, intertidal wetlands

## Abstract

Our aim was to investigate the oviposition and larval habitats of the saltwater mosquito *Aedes vigilax* (Skuse) (Diptera: Culicidae) in a mangrove forest system in subtropical Queensland, Australia. Eggshells (indicators of oviposition) and larvae were sampled in three habitat classes that were depicted in a schematic model. Two classes were in depressions or basins, either with hummocks or dense pneumatophore substrates, both of which retained water after tidal flooding. The third class was in freely flushed mangroves that corresponded with more frequent tidal connections than the depression classes. ANOVA and Tukey-Kramer tests were used to analyze the data. The null hypotheses were rejected: the hummock class was a significant habitat based on both eggshell and larval data. The conclusion was that mosquito production in the mangrove system was distributed unevenly between habitat classes, and that the hummock class had conditions suited to the requirements of the immature stages of *Ae*. *vigilax*. This research has the potential to inform mosquito management strategies by focusing treatment on the problem habitats and underpinning habitat modifications including reducing water retention in the basins.

## Introduction

The saltwater mosquito *Aedes vigilax* (Skuse) (Diptera: Culicidae) is common in saltmarsh and mangrove intertidal wetlands in Australia, and is a vector of mosquito-borne diseases of medical significance (Dale et al. 2008) including Ross River virus and Barmah Forest virus. The insect breeds episodically in large numbers along the coast of sub—tropical and tropical Australia, often in wetlands adjacent to densely populated urban areas. It is thus the focus of expensive control programs, where, in SE Queensland, larviciding is the preferred and usual choice. For larviciding to be effective, control needs to be underpinned by accurate information on the distribution of the immature stage habitat, which requires an understanding of the insect's habitat. In saltmarsh environments, knowledge of specific immature stage habitat has been the focus of much research, particularly by Dale since the mid-1980s (such as Dale et al. 1986; Dale and Hulsman 1990; Dale 2008; Dale et al. 2008). As a result, for control in saltmarshes, options exist that are complementary to chemical approaches including habitat modifications such as runnelling and open water marsh management (Hulsman et al. 1989; Dale and Hulsman 1990; Dale and Knight 2006). Runnelling, unlike other environment—based approaches, involves limiting the environmental impact to the saltmarsh by minimally enhancing tidal connectivity sufficient only for controlling larval production.

Compared with saltmarshes, less is known of *Ae*. *vigilax* habitat use in mangroves. As a consequence, mosquito control is largely limited to chemical use and is usually applied broadly across problem mangrove forest areas. Also, because of the lack of mangrove forest—specific *Ae*. *vigilax* habitat knowledge, it has not been possible to develop environmentally based control approaches comparable to runnelling.

Historically there has been limited interest in *Ae*. *vigilax* habitats in mangroves. This was demonstrated by Lee et al. (1984), who summarized *Ae*. *vigilax* literature from 1850 through to 1980. They identified almost 500 references, of which only a small proportion focused on biology and habitat. Although there has been some historical resistance to the concept of mangroves as habitat for *Ae*. *vigilax* (such as Hamlyn-Harris 1933), generally the importance of *Ae*. *vigilax* production in mangroves is accepted. But, as Lee et al. (1984, pp. 223) concluded, “there is likely to be some specialization of breeding within mangrove zones which is not yet adequately understood”.

In the 1990s, interest in *Ae*. *vigilax* use of mangrove environments resulted in a number of papers on the topic (including: Ritchie and Jennings 1994; Gislason and Russell 1997; Turner and Streever 1999; Webb and Russell 1999) with progress generally made towards demonstrating mangroves as a significant habitat. For example, Ritchie and Jennings (1994) showed that *Ae*. *vigilax* production levels in mangroves were comparable to production in saltmarshes for sites in SE Queensland. Gislason and Russell (1997) surveyed saltmarsh, mud depressions, and mangrove pneumatophore areas at Homebush Bay in Sydney, Australia, near the site of the Sydney 2000 Olympics. They found no eggshells on mangrove pneumatophore samples, whereas the soil from around the pneumatophores produced 45 eggshells/sample compared with > 2400 eggshells/sample from saltmarsh soil. With regards to mangroves at Homebush Bay, they concluded that further study was required to elucidate *Ae*. *vigilax* use of impounded mangrove areas.

Jacups et al. (2009) provided a detailed assessment of *Ae*. *vigilax* production (from larval surveys) in a range of vegetation communities (both human modified and natural) located adjacent to the northern coastline in the City of Darwin in tropical Northern Australia. They found the greatest larval production areas to be associated with drainage areas (with both saltmarsh and mangrove vegetation) and tidally connected reticulate areas (saltmarsh dominant vegetation). Drainage areas often include earth works that create or enhance habitat conditions for *Ae*. *vigilax* (as with Gislason and Russell 1997). Areas dominated by *Avicennia marina* were also significant for *Ae*. *vigilax* production but to a lesser degree than the drainage and saltmarsh areas. However, in terms of mosquito control effort based on aerial application of *Bti* (in water), the most important were the reticulated tidal areas and the mangrove areas.

**Figure 1.**
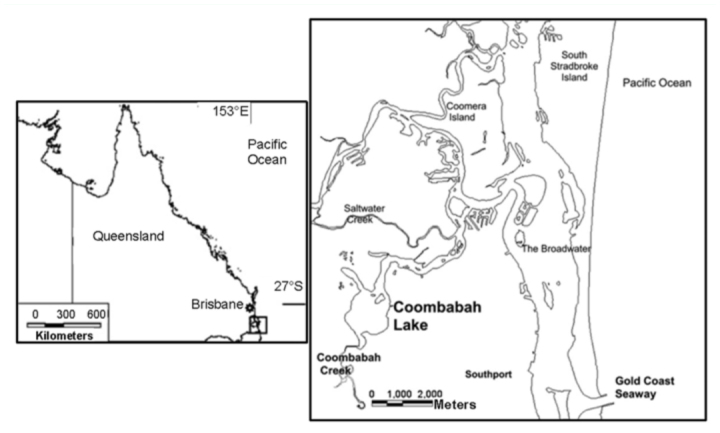
Study site location. High quality figures are available online.

Knight (2008) undertook a detailed study into *Ae*. *vigilax* habitat requirements in the mangroves at Coombabah Lake in SE Queensland, Australia (Figure 1). He established a framework for describing *Ae*.*vigilax* habitat in mangroves involving the interaction of mangrove forest topography, tidal hydrodynamics, and the insect's life cycle. Since then a number of related studies have been published (i.e., Knight et al. 2008; Knight et al. 2009; Griffin et al. 2010) that have examined the underlying drivers related to larval habitat requirements. The current work extends previous knowledge by focusing on *Ae*. *vigilax* habitat within the mangrove forest rather than viewing the mangroves as a unit within the broader intertidal landscape. As a case study, it explores the pattern of immature *Ae*. *vigilax* habitat use within the Coombabah Lake mangroves by relating patterns of substrate structure with eggshell and larval distributions found within the site.

**Figure 2.**
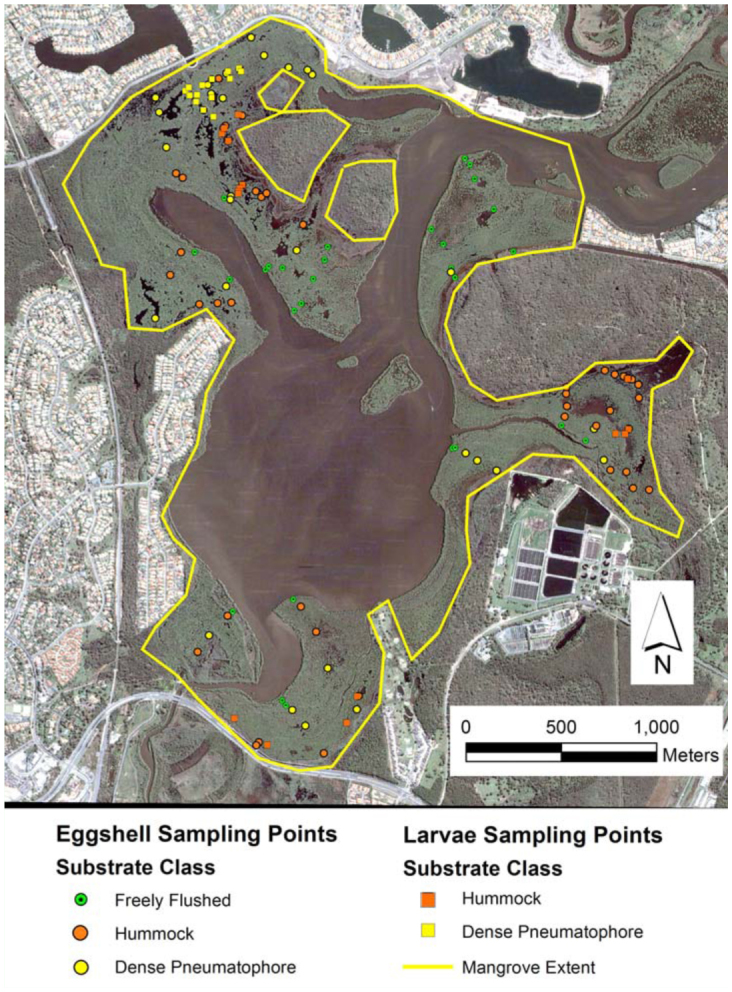
Satellite image (Google Earth 2004) of Coombabah Lake and surroundings showing both the location of *Aedes vigilax* larval and eggshell sampling points with the mangrove forest substrate class identified at each sample point. The extent of mangroves is indicated by the yellow outline. High quality figures are available online.

## Materials and Methods

### Study Area

The study was conducted across the mangrove—forested areas associated with Coombabah Lake (153° 20′ E, 27° 54′ S) (Figure 2), which has been described previously in Knight et al. (2008). In summary, the site is a tidal lagoon wetland connected to the tidal source by approximately six km of creek. The grey mangrove, *Avicennia marina* var. *australasica* (Walp.) (Lamiales: Acanthaceae), is the dominant mangrove tree and, in the basin areas, the only species. Other mangrove species are present on the lake fringes and along creek banks (*Ceriops, Bruguieria*, and *Rhizophora*) but are not dominant. The lake edge is ∼20 km in length and mangroves in the basin areas form bands commonly up to 400 m and as much as 700 m wide. For a detailed description of the basin structures and the system's hydrology see Knight et al. (2008).

### Habitat conceptual model

**Figure 3.**
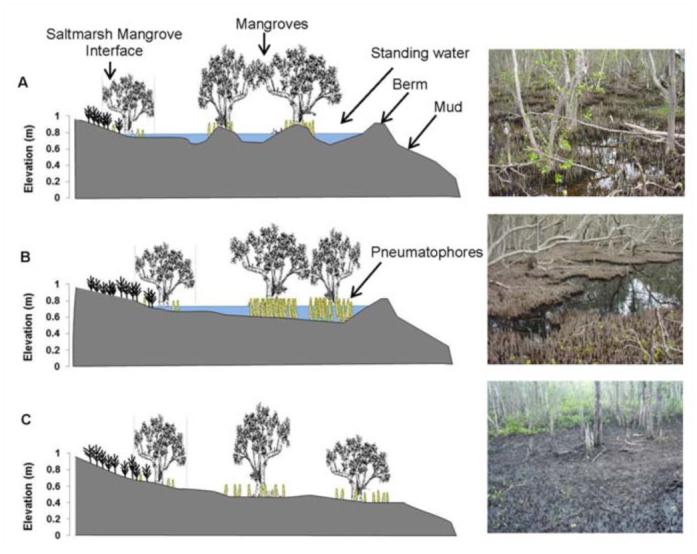
Conceptual model of substrate forms or classes identified at Coombabah Lake (with respect to potential immature *Aedes vigilax*). The vertical axis in each plot is scaled to elevations (Australian Height Datum) recorded across the site, with pneumatophore, water depth and vertical topography to scale. Trees, saltmarsh vegetation, and the horizontal axis are not to scale. The scale of the horizontal axis could range between ∼50 and 700 m. Plot A, B, and C represents the hummock dense pneumatophore, and freely flushed substrate classes, respectively. The left area of each plot shows an area of mixed mangrove and saltmarsh to illustrate a common progression from mangroves into saltmarsh. The tidal flood comes from the right hand edge in each plot. Photos on the far right are indicative of the associated substrate class found at Coombabah Lake. High quality figures are available online.

In order to delineate topographic forms thought to be relevant to mosquito habitat, three substrate forms or classes were identified as shown in the conceptual model (Figure 3). For each class, an area of mixed mangrove and saltmarsh has been drawn to illustrate a common progression from the mangroves into adjacent saltmarsh. The three habitat classes follow.

**Hummock substrate.** (Figure 3A) Composed of mud and some pneumatophores. The hummock substrate was slightly raised (∼0.1– 0.2 m) above standing water (post tidal ebb). The berm has an impounding effect. Water depth ranged between 0.4 and 0.1 m. Pneumatophore density ranged between 200700 m ^-2^ on the hummocks. Individual hummocks were often less than 1 m^2^ and generally spaced < 1 m apart.

**Dense pneumatophore substrate.** (Figure 3B) Composed almost entirely of pneumatophores with densities between ∼1000-2500 m^-2^. The berm has an impounding effect. Dense pneumatophore substrate was usually contiguous over areas of more than 10 m^2^ and exhibited very little micro—topographical variation, as shown in the photo in Figure 3B.

**Freely flushed mangrove substrates**. (Figure 3C) Found in areas where there was no standing water after the tidal ebb, because there was no berm operating to impound tidal water. Freely flushed areas were located either between the tidal source and the basin areas— seaward of a berm—or in areas where there was no lake—edge berm present, with tidal flooding extending up into saltmarsh.

There are some similarities to Provost's (1977) high and low marsh pioneering work in the USA marshes, where the first two classes correspond with mangrove high marshes in Florida and the third class equates to a mangrove form of Provost's low marsh.

### Data collection and analysis

Eggshell sampling was based on the methods of Ritchie and Addison (1991), Ritchie and Jennings (1994) and Dale et al. (1999). Sampling was conducted on 30 July 2005, 25 August 2005 and between 1 December 2008 and 30 April 2009. Two substrate patches (∼ 3 × 3 m each) at 100 sites (Figure 3) were randomly sampled. Sampling at each patch consisted of collecting 15 ×15 cc cores from the top 2.5 cm of substrate. The 15 cores were pooled for each patch and processed in the laboratory following methods of Ritchie and Jennings (1994). The method involves the creation of a water—based slurry (1:1 mix of substrate: water) with a subsample of ∼^⅓^ of the slurry analysed for eggshells. Ritchie and Jennings (1994) reported no significant difference between results from estimating the eggshell density from subsamples compared with the entire sample.

Eggshells were identified as being from one of three species: *Ae*. *alternons* (Linley et al. 1991), notable as being very football shaped; *Verallina funereus* (Linley et al. 1991), cigar shaped; and *Ae*. *vigilax* (Kay and Jorgensen 1986; Linley et al. 1992), between football and cigar shaped based on size and shape. Both hatched and unhatched eggs were counted without regard to eggshell age. Generally, five sampling sites were positioned along transects across a basin area starting near the lake edge/tide source and distributed proportionately landward, with sample points representative of the substrate classes shown in Figure 3. Coordinates for each sample point were extracted from a 2005 Google Earth image of Coombabah Lake, and then loaded into a GPS unit for site location in the field. Where field coordinates varied during sampling new coordinates were recorded.

Larval surveys were undertaken as an initial exploration of *Ae*. *vigilax* production patterns in the Coombabah Lake mangroves with 35 larval surveys conducted in January and February 2005. Surveys were coordinated with local mosquito control personnel to avoid the impact of aerial larviciding. Larvae were sampled using standard 240 mL white (soup ladle type) dipper (Service 1993) on a 1 m handle, by scooping water from around the edge of the substrate in pools while taking care to not disturb larvae. At each site, four samples of water were counted for larvae and summed for each of three randomly selected locations around a site with the three totals averaged to derive a site count. Larvae were counted by slowly decanting water from the ladle by tilting and counting larvae as they spilled from the dipper. There was no attempt to differentiate species or instar in the larval surveys. Larval surveys were conducted at random points in each substrate class (Figure 3) based on access to the site.

A brief description of the substrate composition was noted, including water depth and substrate composition. Both the larval count and eggshell density data fitted a Poisson distribution and were log transformed Ln(x + 1) to approximate normal distributions before being analysed using a one—way ANOVA. Where the ANOVA was significant, a Tukey-Kramer HSD test (for unequal sample sizes) was used to determine significantly different means, based on the general null hypothesis that means were equal, and, where the alternate hypothesis was a two—tailed test, that any or all means were significantly different from each other. The resulting mean and standard errors were transformed back using (Exp(x) - 1) for reporting purposes and relating back to habitat class. Data analysis was undertaken using the statistical package JMP version 5 (SAS Inc.).

## Results

The eggshell density analysis identified a statistically significant difference (*F* = 11.76, df = 2, *96, p <* 0.01) between the three classes; thus, the null hypothesis was rejected. The Tukey-Kramer HSD test (Table 1) identified two significantly different groupings. The hummock class mean (1.145 eggshells cc^-1^) was two orders of magnitude higher than the other class means (0.049 and 0.009 eggshells ce^-1^ for dense pneumatophore and freely flushed, respectively). The range in eggshell densities (Table 1) highlights the heterogeneity of production levels, even in the productive mangrove areas.

**Figure 4.**
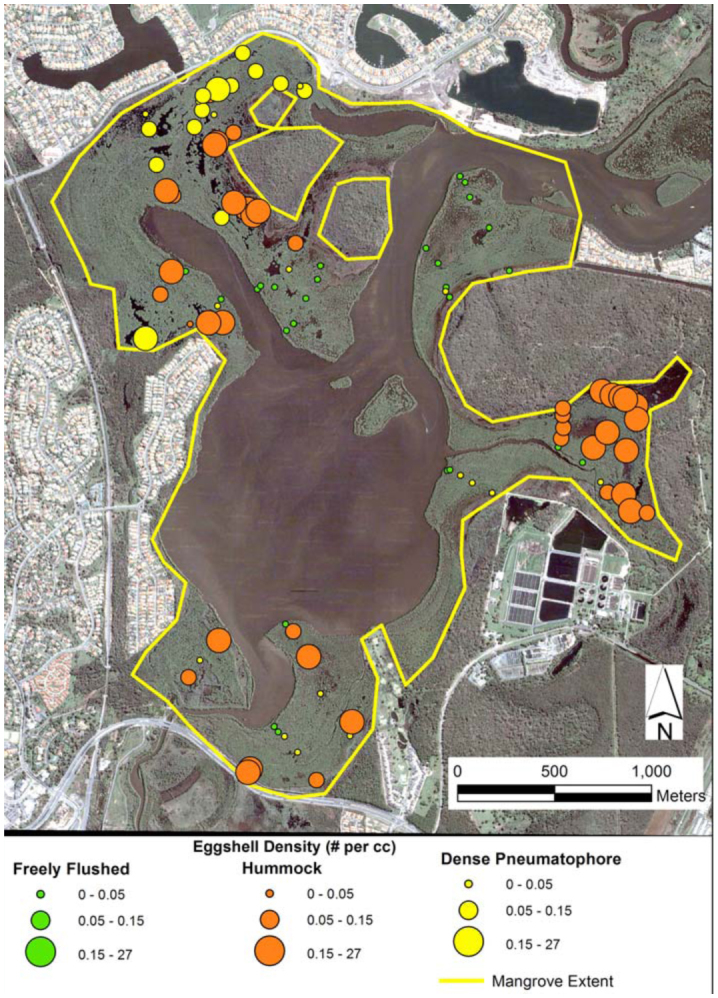
Aerial image of Coombabah Lake showing *Aedes vigilax* eggshell densities grouped by substrate class. The mangrove extent outline shows the area of mangroves potentially treated by aerial control. High quality figures are available online.

**Table 1.**
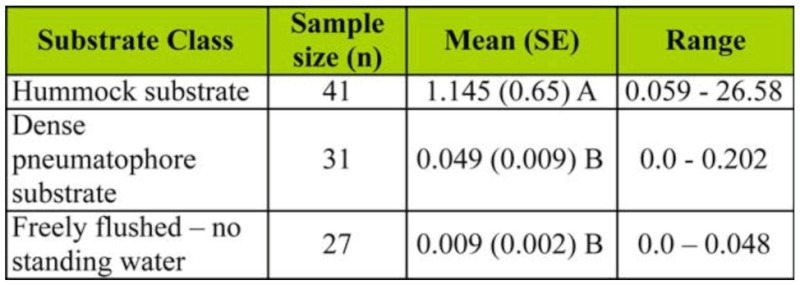
Summary of statistical results from analysis of *Aedes vigilax* eggshell density data (#cc^-1^) collected from within the Coombabah Lake Mangroves.

**Table 2.**
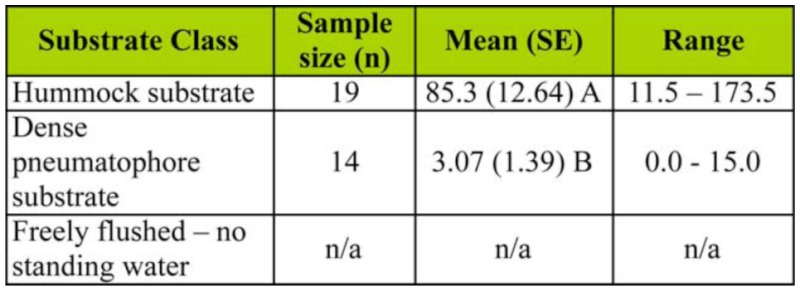
Summary of statistical results from analysis of *Aedes vigilax* larval count data (larvae/dip) collected from within the Coombabah Lake Mangroves.

Analysis of the larval data (Table 2) also enabled rejection of the null hypothesis (*F* = 102.86, df = 1, 31, *p* *<* 0.01). The larval counts in the hummock class (mean = 85.3) were much greater than for the dense pneumatophores (mean = 3.07). Mangrove areas that were freely flushed were not assessed for larval production because of the absence of standing water (see Figure 3).

Results of the eggshell and larval surveys are shown in Figure 4, overlaid onto the image of Coombabah Lake, using a proportionally scaled symbol set with the same color scheme for habitat classes as in Figure 2. Most of the mangrove extent (within the yellow line, Figure 4) is aerially treated by the local mosquito control program.

## Discussion

### Eggshells and substrate class

The highest density of eggshells recorded in the study was 26.58 cc^-1^ from a site with a hummock form (Figure 3A). This is because at this particular site there was a high proportion of smaller mud hummocks (often < 0.4 ×0.4 m), providing an ideal habitat for both ovipositing; additionally, there was a mosaic of standing water for larval habitat. This is consistent with Sinclair (1976), who stated that eggs are laid immediately adjacent to standing water such as edges of pools and drains, or elevated areas within depressions. What is shown here is that although there are different elevated areas in the mangrove depressions, only the hummock form is used as habitat.

The results are consistent with the maximum eggshell densities (cc^-1^) reported in Ritchie and Jennings (1994) of 14.8 for saltmarsh and 11.02 for mangroves in SE Queensland. They are also comparable with Gislason and Russell (1997) who reported a mean of 2428 (SD 2966) eggshell counts per sample (∼40 eggshells cc^-1^) from soil in a *Sarcocornia* saltmarsh at Homebush Bay in Sydney. This demonstrates that the mangrove forest at Coombabah Lake supports the production of high densities of *Ae*. *vigilax.* However, an important question for mosquito control activities is how eggshell densities translate to operational procedures that are based on larval counts; this is discussed below.

### Larvae and substrate class

The only significant larval habitat was the hummock form, where mean larvae/dip were well above the minimum mosquito control larval count level of 5 per dip (Cecily Draper, (Moreton Regional Council, Queensland, Australia, personal communication), used to indicate potential problem levels of *Ae*. *vigilax* production. The same larvae/dip level is used in Florida mangroves (Addison et al. 1992) for *Ae*. *taeniorhynchus* production. At Coombabah Lake the mean larvae/dip count (3.07) for the dense pneumatophore class was below the mosquito control trigger level, suggesting that this substrate class is not a particularly suitable habitat. Two potential reasons for this are the lack of mud in the substrate and unsuitable tidal dynamics (see description of deep basin in Knight et al. 2008) where some of the dense pneumatophore areas at Coombabah Lake experience as few as two tidal connections per year.

### Relationship between eggshells, larvae, and habitat use

Addison et al. (1992) related larval counts with eggshell densities using regression equations for a very similar species (*Ae*. *taeniorhynchus*) prevalent in Florida, USA (for a comparison of *Ae*. *vigilax* with *Ae*. *taeniorhynchus* see Ritchie 1993). Their results were limited by using only a few data points. Also, they suggested that threshold densities of 0.05 eggs cc^-1^ corresponding with 5 larvae/dip were minimum thresholds indicating problem levels of production in Florida mangroves.

In the current work, the mean eggshell density for the hummock class was 1.145 cc^-1^ (Table 1) corresponding with an average of 85.3 larvae per dip (Table 2). Both eggshell density and larval count are well above the thresholds suggested by Addison et al. (1992), demonstrating that the hummock habitat produces *Ae*. *vigilax* well above problem threshold levels for mosquito control agencies.

In contrast, the dense pneumatophore class was found to be a less important habitat with a mean eggshell density of 0.049 cc^-1^, with a correspondingly low mean larval count/dip of 3.07 (Table 2); both of which are below mosquito control thresholds. Also, the results highlight the unsuitability of the freely flushed areas as habitat for *Ae*. *vigilax*, given its low marsh topography (Figure 2C) resulting in relatively frequent tidal connections (see the description of fringing forest in Knight et al. 2008).

A confounding issue is the consequence of some artificial habitat structures, such as ditch banks and vehicle tracks in otherwise non— productive areas. This has somewhat weakened the statistical strength of the research. For example, in an area characterised as dense pneumatophores, a ditchline constructed in the 1940s has produced oviposition sites in an area that would otherwise not be productive. The importance of artificial structures has been identified previously (Gislason and Russell 1997; Jacups et al. 2009; Kurucz et al. 2009).

A second limitation of the research was that the larval data were not differentiated by species, whereas the eggshell data were. It is reasonable that some larvae observed in the dense pneumatophore areas were *Culex sitiens* that oviposit onto the water surface. However, *Cx*. *sitiens* is not a major problem mosquito, and is only more abundant in saltwater habitats after rainfall.

### Extension to operational issues

As an initial indicator of *Ae*. *vigilax* production, the larval survey was informative and supported the eggshell density results. As a mosquito control assessment tool, the larval survey is an efficient approach. However, the appearance of larvae is episodic, whereas assessment using eggshells can be undertaken at any time (Ritchie et al. 1992), providing useful information on potential problem areas. The difficulty is in applying eggshell densities as a reliable indicator to determine whether to control for larval production.

The challenge is in determining what level of eggshell density indicates a problem. This varies depending on factors such as proximity to human populations, extent of the habitat area involved, control strategies, disease risk, and human tolerance of mosquitoes.

The pattern of eggshell densities and larval counts across the different substrate classes demonstrates that not all parts of the mangrove basin are significant mosquito problem areas. For this information to be useful for mosquito control, a detailed map is needed that identifies and delineates the productive areas. Such a map depends on detailed topography as shown in the conceptual model and may be obtained from high resolution LiDAR (e.g., Knight et al. 2009).

There is potential to use detailed maps of mosquito production areas in mangroves to plan alternative forms of control using a similar rationale to that of runnelling in saltmarshes. For example, a pilot project to minimally modify tidal connectivity in another mangrove forest is currently being developed using LiDAR data.

## Conclusion

The current research has produced three main results. The first was to confirm that mangrove basin forests provide habitat for significant numbers of *Ae*. *vigilax.* The second was to demonstrate that the pattern of *Ae*. *vigilax* habitat use within mangrove basin forests can be highly heterogeneous, and requires detailed assessment to accurately inform mosquito control programs. The third was to demonstrate that a landscape of hummocks within a mangrove basin environment is a key habitat form.

This research has (1) contributed to filling the gap in knowledge about mosquito production within mangrove forest systems, (2) dispelled the idea that immature mosquitoes may be distributed evenly in such systems, by rejecting the null hypothesis, (3) confirmed the *Ae*. *vigilax* habitat requirements in mangrove forest demonstrating that the most significant areas are basins with hummocks (larvae and eggshells), areas that are periodically exposed and also inundated, and (4) confirmed that eggshell density can be used to identify productive habitat for *Ae*. *vigilax*.

Further, this research may help reduce costs of mosquito management by (5) reducing the area treated by improving knowledge of the distribution of the significant habitats, indicating areas where control may, or may not, be needed, and (6) showing that there is the potential to modify the problem habitats to reduce their suitability for mosquito production.
